# A benchmark dataset of Primitive Indian Paddy Panicle Images and identification via deep residual transfer learning

**DOI:** 10.1016/j.dib.2026.112774

**Published:** 2026-04-13

**Authors:** Kunal Mishra, Santi Kumari Behera, Prabira Kumar Sethy, Ranjith Pamerelli, Aseel Smerat, Anita Khanna, Aziz Nanthaamornphong

**Affiliations:** a VSSUT Burla, Odisha, India; b Sambalpur University, Burla, Odisha, India; c OUAT Chiplima, Odisha, India; d Al-Ahliyya Amman University, Amman 19328, Jordan; e Guru Ghasidas Vishwavidyalaya, Bilaspur, C.G., India; f College of Computing, Prince of Songkla University, Phuket Campus, Thailand

**Keywords:** Primitive rice, Panicle dataset, Crop phenotyping, ResNet transfer learning

## Abstract

We introduce ``Primitive Indian Paddy Panicle Images,'' a benchmark image dataset of 22 primitive Indian rice panicle varieties (Sethy, Prabira; Pamerelli, Ranjith, 2026; Mendeley Data, V1, doi:10.17632/khfd7pzskd.1) and present an identification approach based on deep residual transfer learning. Using a transfer-learned ResNet-50 with image augmentation and an 80/10/10 train/validation/test split, the model attains 100.0% validation accuracy and 98.74% accuracy on the held-out test set. Per-class one-vs-rest AUCs on validation are 1.000 for all 22 classes; test AUCs range from 0.9924 to 1.000 (mean ≈ 0.999), with separate confusion matrices and ROC curves provided for validation and test partitions. These results demonstrate that deep residual transfer learning can robustly discriminate closely related panicle morphotypes when trained on a carefully curated dataset. We release the dataset to support reproducible research in germplasm identification, varietal purity assessment, and automated phenotyping.

Specifications Table

**Table:** 

Subject	Computer Sciences
Specific subject area	Agricultural Science, Computer Science, Food Processing
Type of data	RGB images (JPEG), Raw, Table, Figure, code snippets, baseline results summary
Data collection	The panicles are collected from field and kept in a carpet of red color. Then Images captured under natural light; Harvesting stages; 22 primitive rice varieties
Data source location	Regional Research and Technology Transfer Station (RRTTS), Odisha University of Agriculture & Technology (OUAT), Chiplima, Sambalpur, Odisha, India
Data accessibility	https://data.mendeley.com/drafts/khfd7pzskd Repository name: Mendeley Data identification number: doi: 10.17632/khfd7pzskd.1 Direct URL to data: https://data.mendeley.com/datasets/khfd7pzskd/1
Related research article	No

## 1 Value of the Data

This dataset offers a manually selected and annotated collection of 22 low-level Indian rice panicle accessions to allow for reproducible benchmarking of computer vision approaches to variety recognition/classification and/or trait-measurement applications. High-quality RGB images are acquired under uniform daylight illumination with a flat red background, allowing for comparison of methods for image-preprocessing, augmentation, segmentation etc. Provided with the dataset are train/test splits that maintain a balanced number of images per class, along with a baseline trained on ImageNet-resized input from the popular ResNet-50 architecture. This allows researchers to quickly reproduce transfer-learning experiments as well as easily compare new models or training methodologies. Users may build on these images to create models such as classifiers or object detectors, or leverage them to build feature extractors that can be used for germplasm recognition and/or varietal purity analysis. Additionally, this dataset may be expanded with external metadata such as molecular or field-level data to enable multidisciplinary research involving crop diversity, phenomics, and germplasm conservation.

## 2 Background

This dataset was assembled with the goal of providing a standardized image dataset for varietal identification and automated primitive Indian rice panicle phenotyping. Harvest-stage panicles of 22 locally-conserved varieties were photographed in the field to document morphological diversity captured under natural lighting conditions against a solid red cloth background, which allowed for consistent contrast for computer vision analysis. Our approach follows existing computer-vision pipelines that use image augmentation and transfer learning; we use ResNet-50 here as an example deep residual network for establishing baseline model performance. Images were collected, labeled, and split according to procedures outlined in our readme to create train/val/test splits for reproducible benchmarking. This dataset serves as companion material to our accompanying paper by providing the original imagery resource as well as the class labels and splits used to report model performance, so that users can readily reproduce model training and evaluation.

## 3 Data Description

**Fig. 1:**
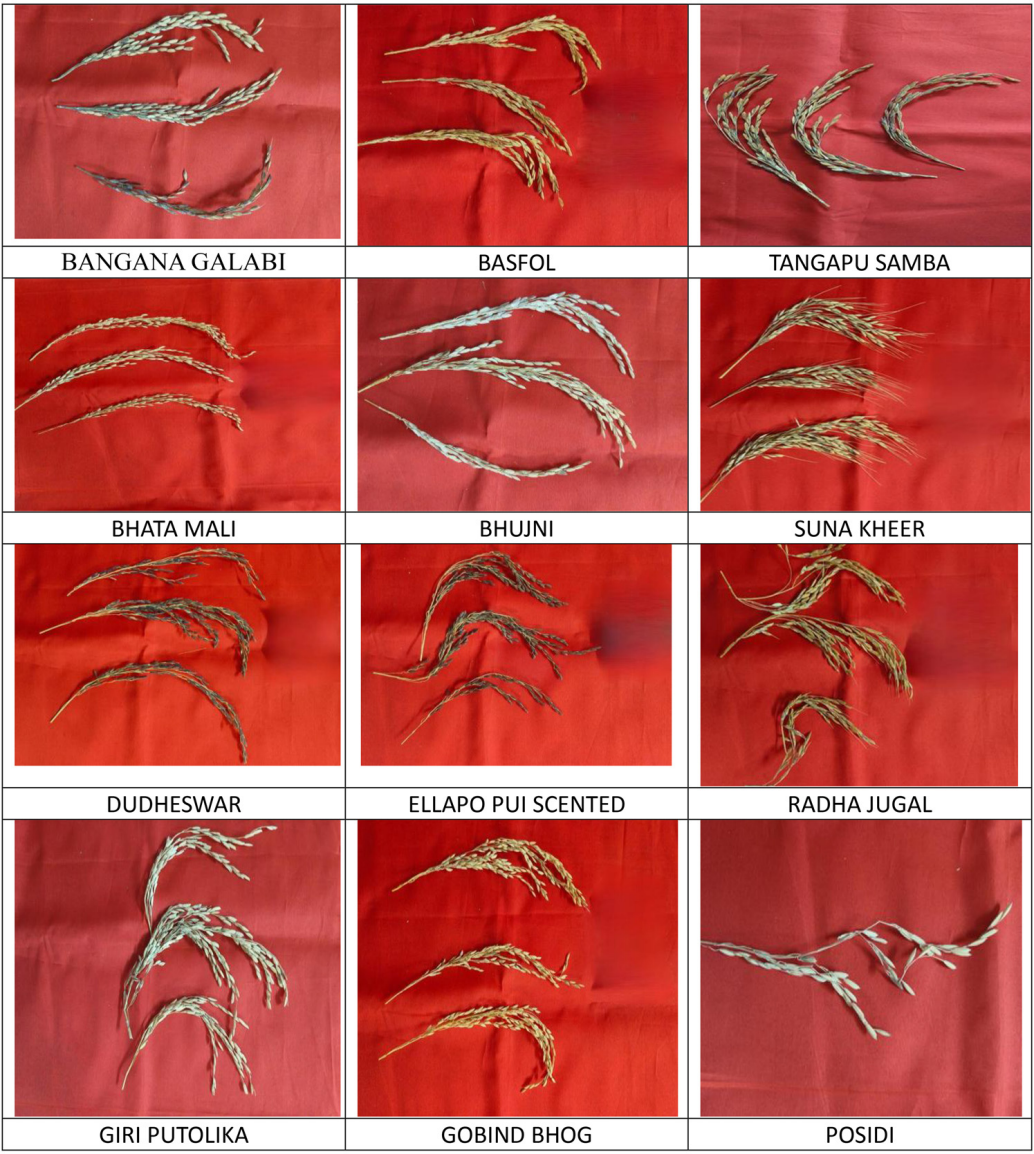

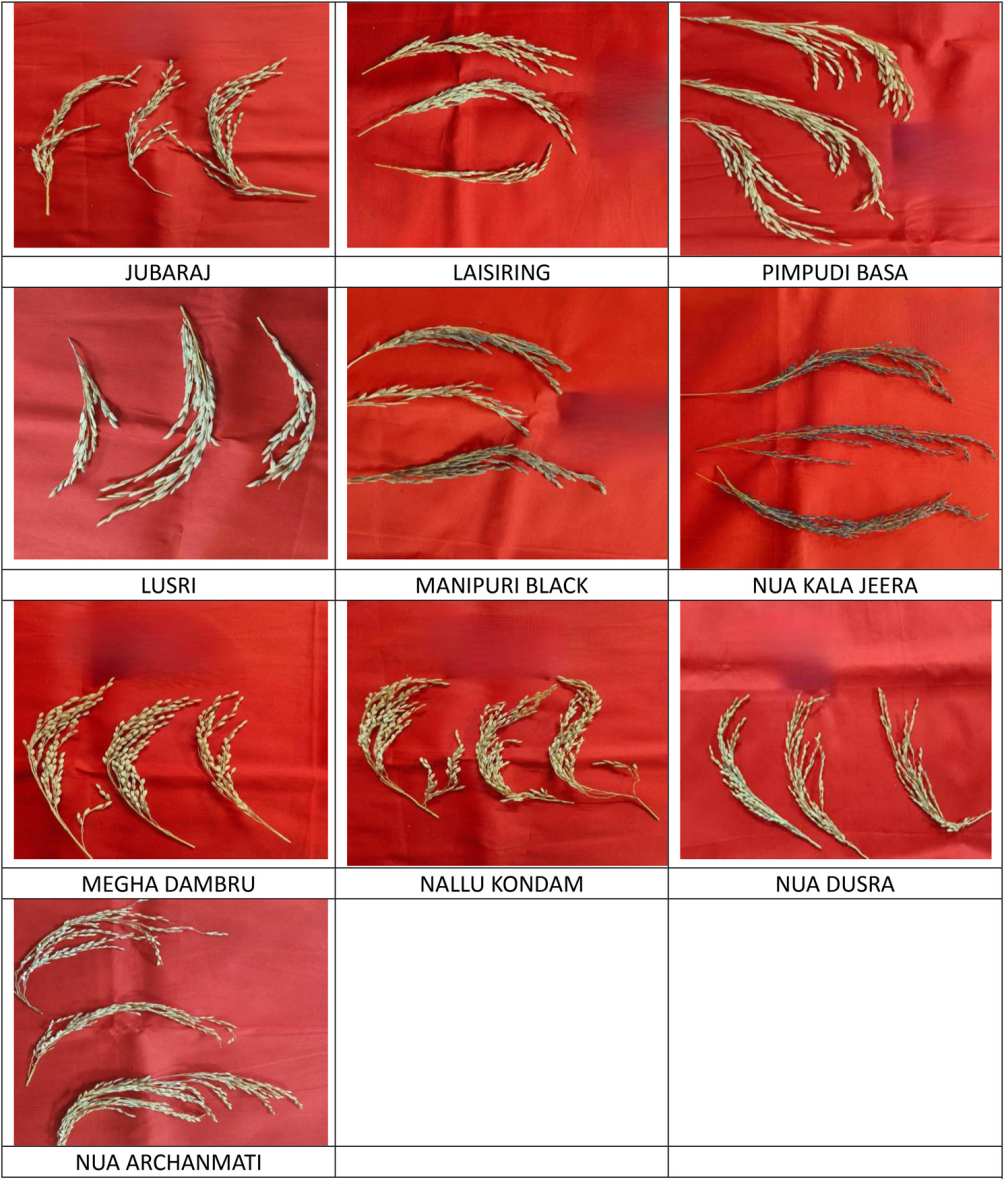
Illustration of 22 varieties of panicle images of primitive paddy.

**Fig. 2:**
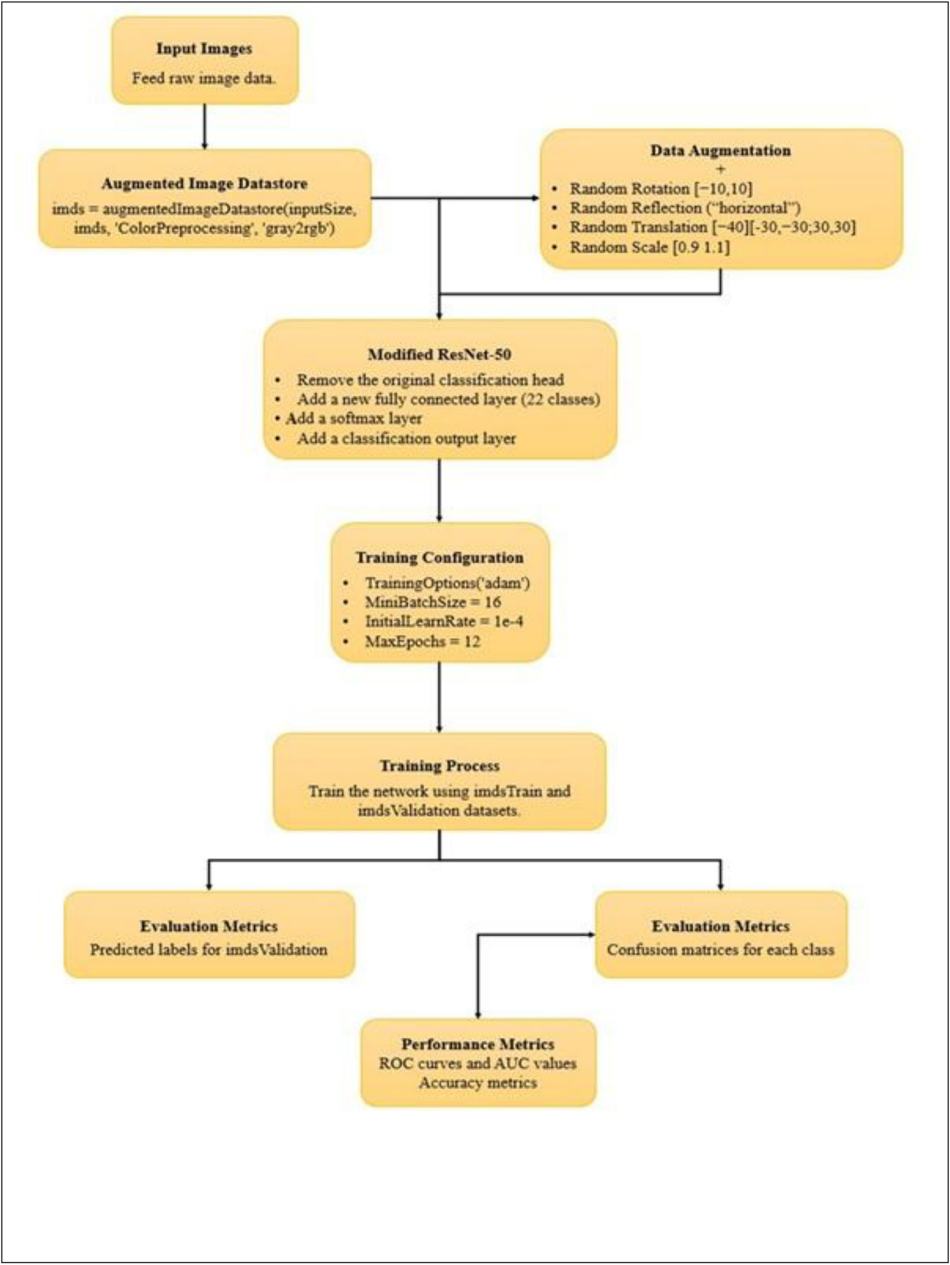
Baseline experiment: Classification of panicle of 22 primitive paddy varieties using modified resnet50.

**Fig. 3:**
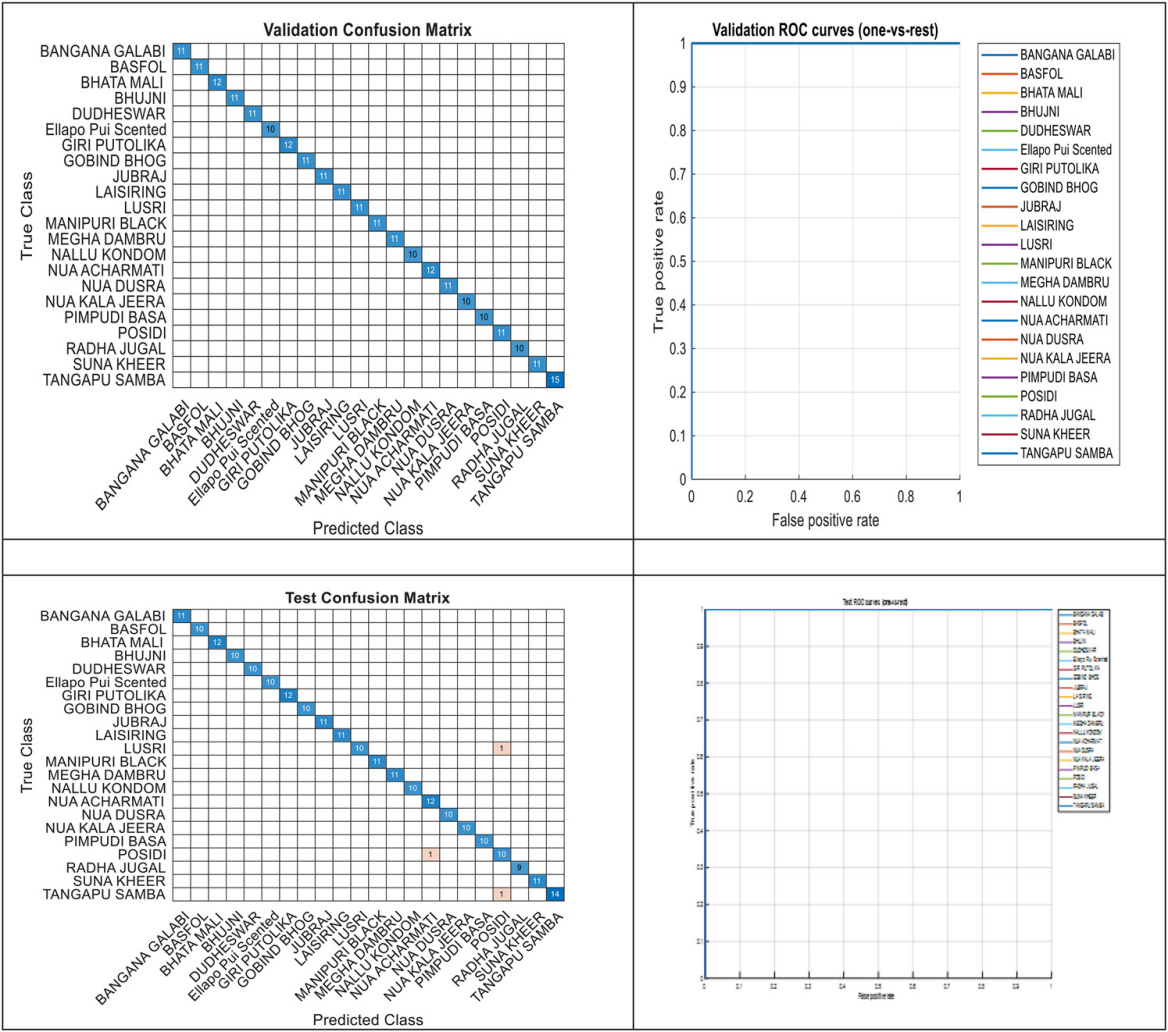
Performance of baseline model (a) validation confusion matrix (b) validation AUC curve (c) test confusion matrix (d) test AUC curve.

The dataset contains 22 classes (panicle images of primitive rice varieties) with approximately 100 RGB field images per class (2411 images total). All images were collected from paddy fields at the Regional Research and Technology Transfer Station (RRTTS), Chiplima, a unit of Orissa University of Agriculture & Technology (OUAT), Sambalpur, Odisha, India. Images are organized into class‑specific subfolders and labeled by folder name. The collection is publicly hosted at the provided Mendeley Data link. sethy, PRABIRA; Pamerelli, Ranjith (2026), “Primitive Indian Paddy Panicle Images”, Mendeley Data, V1, do i: 10.17632/khfd7pzskd.1). The sample of images are illustrated in Fig. 1. The distribution of dataset is illustrated in Table 1 (Figs. 3 and 2).

**Table 1:** Directory structure of dataset.

Paddy Varieties	No. of Images
TANGAPU SAMBA	149
BHATA MALI	122
GIRI PUTOLIKA	118
NUA ACHARMATI	118
POSIDI	111
MANIPURI BLACK	110
JUBRAJ	109
LUSRI	109
MEGHA DAMBRU	108
SUNA KHEER	108
DUDHESWAR	107
BHUJNI	106
BASFOL	105
GOBIND BHOG	105
NUA DUSRA	105
BANGANA GALABI	112
LAISIRING	112
Ellapo Pui Scented	101
NUA KALA JEERA	101
PIMPUDI BASA	101
NALLU KONDOM	98
RADHA JUGAL	96

Here, per-class sample counts vary modestly across the 22 varieties (total = 2411 images). This variation was not intentionally designed but reflects natural availability of harvest-stage panicles in the conserved accessions at the time of collection (field sampling constraints and differing panicle yields per accession). To assist users, we provide per-class counts and summary statistics: mean = 109.6 images/class, standard deviation = 14.2, minimum = 96, maximum = 149. While the dataset is approximately balanced for comparative benchmarking, users should consider the slight class-size differences when training models (e.g., use class-weighting, stratified sampling, or targeted augmentation for smaller classes).

## 4 Experimental Design, Materials and Methods

Harvested panicles of 22 locally conserved primitive varieties of Indian rice were acquired at Regional Research and Technology Transfer Station (RRTTS), OUAT Chiplima, Sambalpur, Odisha India. These panicles were photographed directly against a planar red cloth background that provided high contrast while minimizing background between-image variation using natural daylight that was homogenous across all images [1]. All images were captured using an iPhone 16 Pro at an approximate handheld distance of 50 cm, with 1–3 panicles arranged on a flat red cloth to minimize overlap. Photographs were taken between 11:00 AM and 3:00 PM on sunny days under natural daylight using the phone’s auto exposure/auto white-balance (ISO/shutter speed auto). Images were saved as RGB JPEGs and later resized to ResNet-50 input dimensions for model training. The resulting dataset contains 2411 RGB JPEG images saved in fixed train/test splits across class folders (training dataset ≈96–149 images/varietal class) and is available openly through Mendeley Data (doi:10.17632/khfd7pzskd.1). Images are sorted into variety specific subfolders within each class folder. There are no train/validation/test subfolders for each class. We randomly generate the 80/10/10 split in our experiments using code with stratified sampling to keep partitions balanced with respect to class. Image sizes were normalized to that of a pretrained ResNet-50 input size and ‘augmentedImageDatastore’ object was created with 'imageAugmenter' parameter functionality of randomly rotating between −10° and 10° Again, random translation is applied. i.e., random translation [−40] [−30, −30, 30, 30] means, the image is moved within a specified range of pixels so that the object inside the image appears at slightly different positions. Here, the range [−30, −30, 30, 30] means the image can be shifted up to 30 pixels left, right, upward, or downward randomly. Similarly, [−40] indicates that the image can be translated randomly within ±40 pixels. This technique is mainly used to increase the diversity of training data, improve model robustness, and help the model learn position-independent features [2]. Furthermore, images were scaled by small factors ranging from 0.9 to 1.1 to enhance between-image appearance variability and improve the generalization capability of the model. The head of ResNet‑50 (fc1000, softmax and classification layers) was removed and instead initialized with a new fully connected layer (matching the number of classes, 22) with higher learning‑rate factors, a softmax layer, and classification output layer [3]. The final pooling layer of the network, i.e., avg_pool was fed to the input of the newly connected head [4]. The ResNet‑50 backbone was fine‑tuned end‑to‑end (learning continued through earlier convolutional layers; none were frozen). Training was carried out with augmentation using the augmentedImageDatastore function and the Adam optimization algorithm [5] (mini‑batch size = 16, initial learning rate = 1e‑4) for 12 epochs. The gray2rgb preprocessing step was included to ensure consistency in the input image format during data processing. Although the original dataset primarily consisted of RGB JPEG images, some images were converted to grayscale during intermediate preprocessing steps such as normalization, filtering, or augmentation. Since the pretrained ResNet‑50 model requires three-channel RGB input images, the gray2rgb function was applied to convert any single-channel grayscale images back into three-channel RGB format. This step ensured uniformity across all input images and prevented dimensional mismatches during training and feature extraction. Validation was performed every epoch number that was dynamically calculated based on the training set size. Testing returned predicted labels and score outputs for validation and held‑out test sets, generated confusion matrices to visualize model performance, and calculated per‑class one‑vs‑rest ROC curves and AUC using the perfcurve() function in MATLAB. Overall accuracy and mean / per‑class AUC were reported. The trained network, training parameters and metrics were saved to allow for reproduction of results. Training was performed using GPU acceleration and MATLAB's Deep Learning Toolbox software suite on a workstation with MATLAB R2022a, an NVIDIA GeForce RTX 3050 GPU, Intel Core i7 CPU, and 8 GB RAM.

## Limitations

Limitations of this work include that images were obtained under controlled conditions (uniform natural daylight and a planar red cloth background), which reduces background and lighting variability and may therefore limit model generalization to in‑field or differently lit images with occlusions. Only harvest‑stage panicles were sampled, so classifier performance on other phenological stages is unknown. Although the dataset is moderately sized (2411 images, ≈100 images per class), some classes have fewer samples, which may mask within‑variety morphological variation and limit geographic/genetic diversity. We acknowledge that the near‑perfect validation and test metrics reported in this study primarily reflect baseline performance on this curated dataset; these results should not be interpreted as guaranteed real‑world performance. Model accuracy is likely to decrease on more heterogeneous image collections, and we therefore recommend that future work evaluate robustness on diverse in‑field images, expand sampling across stages and locations, or apply domain‑adaptation approaches to improve generalization.

## Ethics Statement

Current work does not involve human subjects, animal experiments, or any data collected from social media platforms.

## CRediT Author Statement

Prabira Kumar Sethy (Corresponding author) led the study, contributing to conceptualization, methodology, data curation, investigation, software development, formal analysis, visualization, writing of the original draft, responding to reviewer comments, and project administration. Ranjith Pamerelli contributed substantially to data curation and investigation, provided resources, performed validation, and participated in writing — review & editing. Aseel Smerat supported methodology and software implementation, carried out formal analysis and validation, assisted with visualization, and contributed to writing — review & editing. Anita Khanna participated in investigation and data curation, provided resources and project administration support, and contributed to writing — review & editing. Santi Kumari Behera provided supervision, contributed to methodology, secured funding and resources, and contributed to writing — review & editing. Kunal Mishra contributed to manuscript revision and responding to reviewer comments, assisted with validation and formal analysis, and contributed to writing — review & editing. Aziz Nanthaamornphong contributed to manuscript revision and responding to reviewer comments, assisted with software and code updates for reproducibility, and contributed to writing — review & editing.

## Data Availability

Mendeley DataPrimitive Indian Paddy Panicle Images (Original data).

## References

[1] Sangha H.S., Darr M.J. (2025). Influence of model size and image augmentations on object detection in low-contrast complex background scenes. AI.

[2] Purohit S., Suman S., Kumar A., Sarkar S., Pradhan C., Chatterjee J.M. (2021). Comparative analysis for detecting skin cancer using SGD-based optimizer on a CNN versus DCNN architecture and ResNet-50 versus AlexNet on Adam optimizer. Deep Learn. Pers. Healthc. Serv..

[3] Zhu H., Zhu Z., Wang S., Zhang Y. (2023). CovC-ReDRNet: A deep learning model for COVID-19 classification. Mach. Learn. Knowl. Extr..

[4] Ajmi C., Zapata J., Elferchichi S., Zaafouri A., Laabidi K. (2020). Deep learning technology for weld defects classification based on transfer learning and activation features. Adv. Mater. Sci. Eng..

[5] Jais I.K.M., Ismail A.R. (2019). Adam optimization algorithm for wide and deep neural network. Knowl. Eng. Data Sci..

